# First Optimization of Tomato Pomace in Diets for *Tenebrio molitor* (L.) (Coleoptera: Tenebrionidae)

**DOI:** 10.3390/insects14110854

**Published:** 2023-11-01

**Authors:** Ferdinando Baldacchino, Anna Spagnoletta, Flutura Lamaj, Maria Luisa Vitale, Vincenzo Verrastro

**Affiliations:** 1 Laboratory of Bioproducts and Bioprocess, ENEA—Trisaia Research Centre, S.S. Jonica 106, Km 419+500, I-75026 Rotondella, Italy; 2 CIHEAM-Bari, Mediterranean Agronomic Institute of Bari, Via Ceglie, 9, I-70100 Valenzano, Italy

**Keywords:** Yellow Mealworm, edible insects, by-products, rearing substrates, fatty acid, nutraceutical, antioxidant, lycopene, β-carotene

## Abstract

**Simple Summary:**

Rearing substrates based on agri-food by-products are ideal for converting bioactive-rich waste and increasing insect quality. This approach is limited by the need to provide nutritionally balanced diets for farmed insects. In this study, we evaluated the possible use of tomato pomace (TP), an agro-industrial waste from tomato processing, as a component in rearing substrate for Yellow Mealworm (*Tenebrio molitor*). We compared bran-based diets with tomato pomace (0%, 27%, 41%, and 100%). As brewer’s spent grain and yeast, protein sources are used in mixed diets to ensure equal protein contents to the diet control. The results showed no difference in larval performance between diets, except for a longer time of the development in the TP100 diet. Generally, the efficiency indices worsened in diets with increasing TP. Conversely, lycopene and β-carotene increased in the harvested larvae, and the fatty acid composition wasimproved, with an increase in polyunsaturated fatty acids. Maximum qualitative increases were obtained with the TP100 diet. The TP41 diet is the best balance between larval performance and qualitative improvement. Therefore, tomato pomace is suitable for the formulation of mealworm diets, even in high dosages when supplemented with proteins and carbohydrates.

**Abstract:**

Tomato pomace (TP), an agricultural industrial waste product from the tomato processing industry, is valorized as a rearing substrate for *Tenebrio molitor* (L.). This study evaluated bran-based diets with increasing tomato pomace (0%, 27%, 41%, and 100%). Protein sources, such as brewer’s spent grain and yeast, were used in TP27 and TP41 diets to ensure equal protein contents to the control diet. Results showed no different for larval and pupal weights between diets; however, the time of development significantly increases in TP100 compared to all diets. The feed conversion rate progressively increases from 2.7 to 4.3, respectively, from the control to the TP100 diet. Conversely, lycopene and β-carotene increase in the larvae. The fatty acid composition improves by increasing polyunsaturated fatty acids (mainly α-linoleic acid). Although the best nutritional quality was obtained in T100, the TP41 is the optimal diet for balance between larval performance and qualitative improvement of larvae. Therefore, tomato pomace is suitable for the formulation of mealworm diets, even in high dosages, when supplemented with sustainable protein and carbohydrate sources.

## 1. Introduction

The use of by-products as a growth substrate (from here below indicated as “diet”) allows for reduced production costs in rearing edible insects, and increases their role as “bioconverters” in the circular economy [1,2]. Moreover, this technique further reduces the production of greenhouse gases, already low by farmed insects compared to livestock [3]. This approach is mainly focused on the Black Soldier Fly (*Hermetia illucens* L.; Diptera: Stratiomizidae) [4], but can also be transferred to Yellow Mealworm (*Tenebrio molitor* L.; Coleoptera: Tenebrionidae) with the choice of suitable by-products and the correct formulation of diets. This choice must also consider the bioaccumulation capacity of heavy metals, pesticides, and various pollutants by farmed insects [5,6,7,8]. The diet affects the growth performance of *T. molitor* larvae or mealworms (MLW) [9] and the productivity of their adults [10]. The diet also impacts the nutritional composition of collected larvae [9,11]. The latter ability has frequently been thought to enhance the protein content and amino acid composition [12]. Diet affects the fatty acid (FA) composition [9,13,14] and the nutritional quality indices [15,16,17].A diet high in linseed has been shown to increase polyunsaturated fatty acids (PUFA) [15]. Diets have also been shown to improve calcium content and produce a beneficial Ca:P ratio [11,18,19].

Recently, the interest of some researchers has focused on increasing antioxidants in larvae through the diet supplemented with specific agricultural by-products. Some tested by-products were orange peels [16], mastic and olive leaves, and waste tomato (peels and seeds) [20].

This approach is limited by the need to provide balanced diets for the farmed insects’ nutritional requirements.

World tomato production is estimated at 189 million tons in 2021 [21] and 39 million tons of processed tomatoes per year, estimated by the Word Processing Tomato Council [22].

Tomato waste can be used for the production of biofuels [23], but tomato pomace (TP) is receiving attention to recover valuable components (such as tomato seed oil, protein, lycopene, dietary fiber, etc.) [24]. Seasonal availability (typically summer) and the shelf-life of TP can be increased by preliminary drying, preferably sustainable [25]. Tomato pomace consists of 5–10% of the fresh weight of tomatoes [26], and it is made of peels, seeds, and residual pulp [27]. The nutritional composition is influenced by the proportions of the different components and by the transformation process. Generally, its composition is made up of fiber (53.0%), sugars (25.7%), protein (19.3%), and fat (5.9%) on a dry weight basis [28]. It is also a source of carotenoids, especially lycopene and β-carotene in the peel [29].

Classified among the phytochemicals, carotenoids, as well as polyphenols [30], show marked antioxidant [31] and anti-inflammatory activity [32].

Due to these properties, several studies have associated their use with multiple health benefits, in particular α- and β-carotene and β-cryptoxanthin as a valuable source of vitamin A [33], lutein and β-carotene as positive adjuvants in many eye-related diseases, including cataracts and age-related macular degeneration [34], and finally lycopene and β-carotene as skin protectors from UV rays [35], adjuvans in the prevention of cancer [36] and heart health [37].

The inclusion of tomato pomace in animal nutrition has been tested [38] and evaluated on poultry [39], quail [40], ruminants [41], dairy cows [42], and lamb [26].

The use of this by-product to feed insects has been little investigated, although recently, tomato was tested by *H. illucens* [43], and TP was included as a supplement (10% *w*/*w*) in diets for *T. molitor* [44]. A greater supplement would alter the initial nutritional composition of the diet; therefore, high-dose tomato pomace requires the formulation of specific isoproteic diets.

This work aims to evaluate four diets, assembled with increasing doses of dried tomato pomace and different protein sources, evaluating its influence on the larvae’s growth performance and nutritional quality to optimize their diet.

## 2. Materials and Methods

### 2.1. Yellow Mealworms Colony

Mealworms used in this work were reared at the *insectarium* of CIHEAM-Bari (in the Apulia region). In rearing, *T. molitor* adults in oviposition were fed a bran-based diet and yeast (ratio 95:5).Subsequently, the same boxes were periodically supplemented with only bran based on the diet eaten by the growing larvae. The wet supplement was distributed twice a week with pieces of organic pumpkin (*Cucurbita moschata*, cv. Butternut) cultivated at CIHEAM-Bari fields. Rearing and subsequent experiments were conducted in a climatic room maintained at 28 ± 1 °C, 70 ± 5% RH, and 0L:24D photoperiod.

### 2.2. Substrate Composition/Preparation

Four different by-products were used to formulate the tested diets. A local organic farm (“Antica Enotria”, Cerignola, Italy) supplied tomato pomace as a by-product of the production of tomato sauce. The pomace consisted mainly of peels and seeds, while the presence of pulp was scarce. Bran was purchased from the mill (Molino “Cimminelli”, Montegiordano, Italy) and derived from durum wheat milling. Brewer’s spent grain was supplied from small local brewers (Brewery “Jazz Beer”, Bernalda, Italy). Zootechnical yeast, as a protein supplement, was purchased from Zabele Srl (Padova, Italy). By-products were preliminarily dried at 60 °C for 24 h in a food dehydrator (COSORI, mod. CP267-FD-RXS, Anaheim, CA, USA). Subsequently, matrices were sieved using a 2 mm manual sieve, and the coarse part was ground, ensuring homogeneity to avoid the influence of particle size [45]. The nutrient composition was determined on a representative sample using AOAC methods [46] (Table 1), and further methods will be described below.

Diets were formulated with bran, different doses of tomato pomace, and brewer’s spent grain and yeast (Table 2). The control diet (TP0) was composed exclusively of wheat bran [9,47,48] and according to dietary supplementation used in CIHEAM-Bari *insectarium* for the growth phase of the larvae. Two other isoproteic diets were formulated by integrating bran with tomato pomace and brewer’s spent grain (TP27) or with tomato pomace and yeast (TP41). Finally, a diet with only tomato pomace (TP100) was tested. Table 2 reports the macro-nutrient and energy values (calculated by conversion factors in Regulation (EU) 1169/2011, Annex XIV). All diets were assembled in the form of “cookies” (5 g of each replica) to avoid self-selection in mixed diets [49] and to facilitate the separation of an uneaten diet (but aggregated in “cookie”) from frass through the sieve (0.5 mm). The “cookies” were obtained by integrating and modifying previous procedures [12,50]. The homogenized by-products were kneaded with Carrageenan 1% (*w*/*w*) and water. The diet was rolled out in a thin layer and dried in an oven at 60 °C for 24 h to ensure shelf-life.

### 2.3. Experimental Set-up

At the beginning of the experiment, six-week-old larvae (28 ± 1 mg) were distributed in groups of 20 larvae [47] in plastic cups (bottom diameter 6 cm). Larvae were fed *ad libitum* with their respective diets, and wet supplements were provided twice weekly. Complete randomization was applied to the experimental design, with 10 replicates/treatment and 20 larvae/replicate.

For each replicate, the weight of the collected larvae, the residual substrate, and the frass were determined with an analytical scale (Mettler-Toledo, mod. B2002-S; precision ± 0.1 mg). The collected larvae were starved for 48 h, blanched at 100 °C for 5 min, and dried at 60 °C for 24 h in a food dehydrator by Purschke et al. [51]. The adoption of these specific pre-treatments and drying methods guarantees the obtaining of flours with the lowest degree of browning due to the action of the phenol-oxidase enzyme and greater stability of the nutritional component [51,52,53,54,55]

Dried larvae, substrate, and frass were stored at −18 °C and powdered before chemical analysis. The latter was performed on three replicas per diet.

### 2.4. Mealworm Growth Perfomance

At the formation of the first pupa (time of larvae collection), the replica was stopped, and larval weight (mg) and pupal weight (mg) were measured. At the same time, larval survival (Equation (1)) and time of development (Equation (2)) were calculated by:

Larval survival (%) = n. initial larvae/n. final larvae and pupae × 100(1)

Time of development (d) = n. days between start of experiment and emergence of first pupa(2)

The efficiency indices were calculated by feed consumption (FC) (mg larvae^−1^), excluding the wet supplement, and by the equations described below:

Feed Conversion Rate (FCR) = FC/WG(3)

where WG represents the larval gained weight at the end of the experiment;

Specific Growth Ratio (SGR) (% day^−1^) = 100 × (lnFW − lnIW)/days (4)

where FW and IW represent the final and initial fresh larval weight;

Efficiency of Conversion of Ingested feed (ECI) (%) = [WG/FC] × 100(5)

Efficiency of Conversion of Digested feed (ECD) (%) = [WG/(FC − Frass)] × 100(6)

were calculated considering WG, FC, and frass as dry weights [56].

### 2.5. Carotenoids Analysis

Carotenoids were extracted from feed, mealworms, and feces. According to Leni et al. [43], extraction was conducted with some modifications. A total of 400 mg of homogenized sample was mixed with 10 mL of hexane/ethanol/acetone (50:25:25) extraction mixture containing 0.1% (*w*/*v*) l-ascorbic acid. After stirring at 200 rpm for 1 h on ice and under subdued light (Universal Table Shaker 709), the samples were centrifuged at 2800 rpm at 4 °C for 20 min (Heraeus Laborfuge 400R–Thermo Fisher Scientific, Asse, Belgium), and the supernatant was separated from the pellet. The separation and quantification of lycopene and *β*-carotene were conducted according to Anthon and Barret [57], with some modifications. Distilled water (1.5 mL per 10 mL of extract) was added to the extract to cause phase separation. After stirring for 1 min under subdued light, the samples were centrifuged at 2800 rpm at 4 °C for 10 min, and the upper hexane phase was recovered and used for spectrophotometric carotenoid quantification (Multiscan Go Spectrophotometer). Samples were read at 503 and 444 nm. The concentration of lycopene and β-carotene was calculated using the following Equations (7) and (8):

Lycopene (mg/kg) = (6.95 × Abs503 nm − 1.59 × Abs444 nm) × 0.55 × 537 × V/W(7)

β-Carotene (mg/kg) = (9.38 × Abs444 nm − 6.70 × Abs503 nm) × 0.55 × 537 × V/W(8)

where:

- 0.55 = the final hexane layer volume ratio to the volume of mixed solvents added for hexane:acetone: ethanol (2:1:1);
- W (mg) = the weight of sample analyzed;
- V (mL) = the volume of mixed solvents added;
- 537 = the molecular weights of lycopene and β-carotene (g/mol);
- 6.95, 1.59, 9.38, and 6.70 = numerical coefficients obtained by solving the Lambert–Beer equations, which link the value of the concentrations of lycopene and β-Carotene to the absorbance and the respective ε (molar extinction coefficients) to the two established wavelengths (444 nm and 503 nm) [57].

### 2.6. Lipid Analysis in Mealworms

The defatting process of mealworm powder (MLWP) was performed according to Gkingali et al. [58] with some modifications. In brief, a three-step extraction procedure using n-hexane removed the fat and any brown lipophilic melonoidins from the MLWP [59]. The sample was first mixed with n-hexane at a ratio of 1:5 (*w*/*v*). The mixture was then shaken for 1 h at 150 rpm (25 °C) using a rotary shaker (Universal Table Shaker 709). After centrifuging the resultant slurry at 8500 rpm for 10 min at 10 °C, the organic phase in the supernatant was decanted. The supernatant was separated from the sediment and stored separately. The process was repeated twice, adding more hexane to the sediment each time. Supernatants were collected in a pre-weighed round-bottom flask, and the n-hexane was evaporated using a rotary evaporator (Rotary Evaporator InstrumentsKentron-Strike 202, Steroglass s.r.l., Perugia, Italy). The final sediment was left at room temperature to eliminate any remaining solvent. The resultant defatted larvae powder (DLP) and lipid extract were kept in a freezer at −18 °C until used. The oil extraction yield (or crude fat %) was calculated according to Equation (9) [60]:

Oil extraction yield (%) = [mass of extracted fat(g)/solids of the initial sample (g)] × 100(9)

Lipids extracted from MLWP were directly trans esterified by producing fatty acid methyl esters (FAME) by applying the technique described by Tasselli et al. [61]. The separation of FAMEs was carried out using an Agilent GC7890A gas chromatograph fitted with a split–splitless injector and a flame ionization detector (FID) at the settings specified by Di Fidio et al. [62]. The retention times of the fatty acids were compared to FAME standards (Merk Life Science S.r.l, Milano, Italy), and their percentage was estimated using the combined area of the present peaks.

### 2.7. Nutritional Quality Indices

The fatty acid profile data were processed, deriving the following nutritional quality indices.

The atherogenicity index (IA) evaluates the atherogenic potential of FAs by relating the total quantity of saturated fatty acids (SFA) and the total quantity of unsaturated fatty acids (UFA) present in the matrix/food. Except for stearic acid (C18:0), SFAs are considered pro-atherogenic, as they encourage lipids to adhere to cells of the immune and circulatory system [63]. UFAs, on the other hand, are attributed to an antiatherogenic effect as they prevent plaque formation and lower cholesterol, phospholipids, and fatty acid esterase levels [64].

The thrombogenicity index (IT) evaluates the tendency to form blood clots in blood vessels. It correlates the pro-thrombogenic FAs, in particular lauric acid (C12:0), myristic acid (C14:0) and palmitic acid (C16:0), and anti-thrombogenic monounsaturated fatty acids (MUFA) and PUFAs ω-3 and ω-6 [65].

Indices of atherogenicity (IA) and thrombogenicity (IT) were calculated using Equations (10) and (11) previously described by Ulbricht and Southgate [66]:

(10) $$IA=\frac{C12:0+\left(4\;\times \;C14:0\right)+C16:0}{PUFA(\sum n-6+\sum n-3)+C18:1+\sum MUFA}$$

(11) $$IT=\frac{C14:0+C16:0+C18:0}{\left(0.5\;\times \;C18:1\right)+(0.5\;\times \;\sum MUFA)+\left(0.5\;\times \;n-6\right)+\left(3\;\times \;n-3\right)+\frac{n-3}{n-6}}$$

The hypocholesterolemic/hypercholesterolemic (HH) ratio characterizes the relationship between hypocholesterolemic and hypercholesterolemic FAs. The HH ratio may more correctly depict the impact of the FA composition on cardiovascular disease than the PUFA/SFA ratio [67], and was calculated as reported by Santos-Silva et al. [68] using the following Equation (12):

(12) $$HH=\frac{(C18:1,cis-9)+\sum PUFA}{C14:0+C16:0}$$

The unsaturated index (UI) indicates the degree of unsaturation of the lipid component and is calculated as the sum of the percentage of each UFA multiplied by the number of double bonds contained by the various FAs. This index more fully reflects the proportion of UFAs with different degrees of unsaturation in the total composition of FAs present in the sample and their influence on the degree of fluidity of cell membranes [69,70]. The UI was calculated using the following Equation (13) previously described to Chen et al. [67]:

UI = [(1 × %monoenoics) + (2 × %dienoics) + (3 × % trienoics) + (4 × % tetraenoics) + (5 × % pentaenoics) + (6 × % hexaenoics)](13)

The calculated oxidability (Cox) value analyzes the fatty acid composition’s effect on the lipid fraction’s oxidative stability. In particular, it evaluates the percentage of C18 UFAs in the matrix/food [71]. The calculated oxidizability (Cox) index was calculated using Equation (14) previously described to Fatemi et al. [72]:

(14) $$Cox\;Index=\frac{\left(1\;\times \;C18:1\right)+\left(10.3\;\times \;C18:2\right)+(21.6\;\times \;C18:3)}{100}$$

### 2.8. Protein Analysis

Alkaline protein extraction was performed on the DLP samples using the protocol made by Zhao et al. with minor modifications [73]. One g of sample was treated with 15 mL of 0.250 M NaOH at 40 °C under agitation for 1 h in a thermostatic orbital shaker (model 420 series Forma, Thermo Fisher Scientific, Asse, Belgium) and centrifuged at 8500 rpm for 30 min at 4 °C. The extraction procedure was repeated three more times in total. The alkaline conditions applied in this type of extraction have the advantage of reducing any browning of the extract obtained [74]. The supernatant and gel layer from all extractions were pooled and used for the quantification.

The determination of crude protein content was performed according to Kotsou et al. [16] with modifications. In total, 10 µL of the appropriately diluted supernatants pooled was transferred to the wells of a 96-well plate, and 200 µL of diluted Bradford Reagent was added and then shaken for 30 s in a plate reader. The plate was incubated for 10 min at room temperature in the dark. The absorbance was measured at 595 nm with a Multiscan Go Spectrophotometer. A standard calibration curve was prepared using bovine serum albumin. Due to the selective operating conditions adopted and the dilutions made to the sample, the extracts obtained generated a background measurement provided by the sample <1%, a negligible overestimate for a possible correction of the protein values obtained.

### 2.9. Statistical Analysis

Larval performance data were initially submitted for normality and homogeneity of variance tests. A one-way ANOVA was applied at FC, FCR, SRG, and ECI values, followed by a Tukey–Kramer HDS test post-hoc to identify the differences between the diets. Alternatively, the non-parametric Kruskal–Wallis test and pairwise multiple comparisons with Bonferroni correction were applied to the other measured parameters. Significance was assumed at *p* < 0.05. All data were statistically processed by SPSS software version 26.0 (IBM Corporation, Armonk, NY, USA). The three replicas’ qualitative and quantitative analysis data are shown as the mean values ± standard deviation (SD).

## 3. Results

### 3.1. Larval Performances

**Figure 1:**
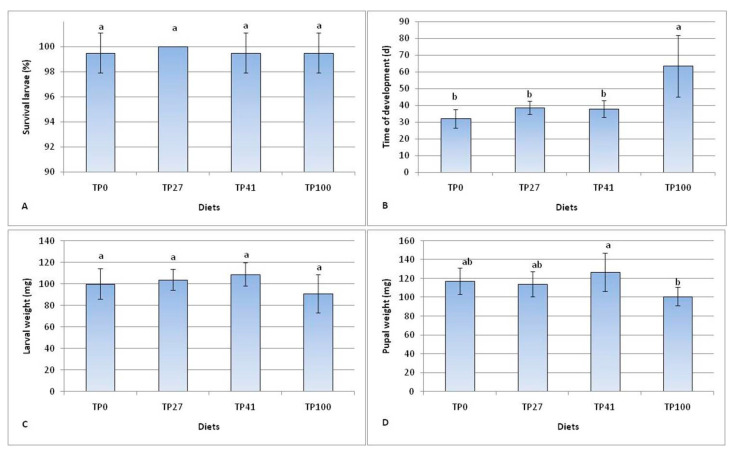
Larval performances on different diets: (**A**) Survival larvae; (**B**) Time of development; (**C**) Larval weight; (**D**) Pupal weight. TP0 as control. The mean ± standard deviation (*n* = 10) with the same letter is not significantly different at α = 0.05 (ANOVA and Tukey–Kramer HDS test).

Larval survival was close to 100% in all diets tested. No statistically significant differences were found between diets (*H* = 1.05; df = 3; *p* = 0.788), including the tomato pomace diet (TP100) (Figure 1).

Values of larval time of development showed significant differences between diets (*H* = 21.23; df = 3; *p* < 0.000). The presence of tomato pomace increased the time of development by 5–6 days in the TP27 and TP41 diets, but these longer periods were not significantly different compared to the 32 days of the control diet (TP0). In contrast, the TP100 diet recorded times of development double (+33.4 days) that of the control and significantly longer than the other diets.

At harvest, the mean larval weight was not significantly different between diets (*H =* 6.41; df = 3; *p* = 0.093). The lowest weight (91.0 mg) was achieved by the TP100 diet (although on larvae collected later), and the larvae of the TP41 diet achieved the highest weight (109.0 mg). The analysis of the weights of the first pupa showed significant differences between diets (*H* = 12.61; df = 3; *p* < 0.006), although significant differences were found only between the TP41 (127.0 mg) and TP100 (101.0 mg) diets (Figure 1).

### 3.2. Efficiency Indicators

Generally, the utilization efficiency of the tested diets decreased with increasing doses of tomato pomace in the diets. The feed consumption was significantly different in the presence of tomato pomace (*F* = 7.8; df = 3, 36; *p* < 0.001) and significantly higher in TP41 and TP100 than in TP0 (Table 3). The feed conversion rate was significantly different between diets (*F* = 73.2; df = 3, 36; *p* < 0.001) with minimum values (FCR= 2.7) in the control, without tomato pomace, and progressively increasing until reaching the maximum in T100 (FRC = 4.3) (Table 3).

The specific growth ratio significantly differed between diets (*F* = 34.1; df = 3, 36; *p* < 0.001). The highest values were obtained with the control diet (4.9%), while slightly lower values were recorded with the two diets mixed with tomato pomace (TP27 and TP41). The TP100 diet achieved significantly lower values (2.5%).

The efficiency of conversion of ingested feed significantly differed between diets (*F* = 85.8; df = 3.36; *p* < 0.001). The ECI values decreased significantly as the amount of tomato pomace increased in the tested diets (Table 3).

The efficiency of conversion of digested feed significantly differed between diets (*H* = 27.3; df = 3; *p* < 0.001). There was no significant difference between the TP0 and TP41 diets, while the TP27 diet showed significantly higher ECD values (42.8%). Finally, significantly higher values than all other diets were obtained in the TP100 diet, with an ECD of 65.9% (Table 3).

### 3.3. Lycopene and β-Carotene Quantification

The values of carotenoids, lycopene and β-carotene, present in feed (diets), stored in mealworms, and excreted with frass, are shown in Table 4. In feed, the addition of tomato pomace shows a proportional increase in the amount of lycopene and β-carotene compared to the control: from 2.66 ug/g TP0 to 179.75 ug/g TP100 for lycopene, from 0.30 ug/g TP0 to 241.5 ug/g TP100 for β-carotene. Furthermore, an inversion of the lycopene/β-carotene ratio between feeds is also evident. While the TP0 diet shows a lycopene/β-carotene ratio >1 (8.8), all the supplemented feeds (TP27, TP41, and TP100) show a value <1 (~0.5). Values referring to the larvae highlighted a general tendency toward accumulating both analyzed carotenoids. In the TP41 and TP100 diets, compared to TP0, there are incremental signals of both lycopene (0.61 ug/g and 1.19 ug/g against 0.08 ug/g) and β-carotene (2.56 ug/g and 7.28 ug/g versus 1.43 ug/g). However, the larvae are richer in β-carotene than in lycopene. In larvae, the increase of both carotenoids is evident only at TP27 (maximum at TP100), unlike the progressive increase observed in diets. The lycopene content in frass varies from 0.70 ug/g in the TP0 to 39.67 ug/g in the TP100, while the β-carotene content varies from 12.09 ug/g in the TP0 to 147.46 ug/g in the TP100; so as seen for the larvae, the frass is richer in β-carotene than in lycopene.

### 3.4. Larval Nutritional Value

**Figure 2:**
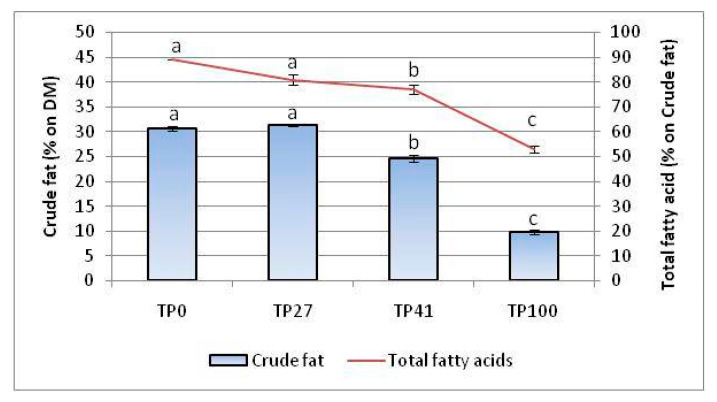
The fat and fatty acid content in mealworms fed on different diets. TP0 as control. The mean ± standard deviation values (*n* = 3) with the same letter are not significantly different at α = 0.05 (ANOVA and Tukey–Kramer HDS test).

Figure 2 shows the fat extracted (% *w*/*w* of MLW powder) from larvae fed different diets enriched with tomato pomace (from 27 to 100%). In our results, the control larvae (TP0) contained 30.76% crude fat. Adding tomato pomace to the feed in percentages of 27% (TP27) does not generate any difference compared to the control diet. However, when tomato pomace was present at 41% (TP41) and 100% (TP100), there was a decrease in the fat content of the larvae. Specifically, TP41 larvae contained 19.6% less fat than control larvae, while TP100 larvae contained as much as 67.9% less fat. Total fatty acid content (TFA) analysis also shows a decreasing value trend compared to TP0 (81%). In particular, TP41 larvae contained about 77% TFA, while TP100 contained 53%, following the trend of crude fat.

The fatty acid composition of mealworms shows seven fatty acids detected and measured in all treatments (Table 5). In TP0 larvae, the main unsaturated fatty acids found were oleic acid (OA) (50.2%), followed by linoleic acid (LA) (25%), while of the saturated fatty acids (SFAs), the most abundant was palmitic acid (PA) (15%). While introducing a diet supplementation of tomato pomace produced no significant change in the amount of SFAs compared to the control, it produced a significant qualitative and quantitative change in UFAs. In fact, in diets TP27, TP41, and TP100, there was a decreasing trend in the percentage of OA, which fell from 50% (TP0) to 26%, and a simultaneous significant increase in PUFAs. The content of LA increases from 25% (TP0) to 40%, while that of linolenic acid (ALA) varies greatly from 0.4% to 2.7%. The PUFA:SFA ratio, calculated to assess our sample’s cardiovascular health benefits, is higher in all three case studies (TP27, TP41, and TP100) than in the control TP0 larvae (1.18). Their PUFA:SFA ratio shows an increasing trend, with values between 1.4 (TP27) and 2.3 (TP100). The influence of diet on the ω-6/ω-3 ratio highlights that all larvae fed with tomato pomace supplementation significantly reduced their ω-6/ω-3 ratio. The greatest 70% reduction was obtained with the TP100 diet, followed by 46% of the TP41, compared to the TP0, which had an ω-6/ω-3 ratio of 64.3.

### 3.5. Lipid Quality Indices

Diet had a direct influence on both the Cox index and UI. As shown in Table 6, in all cases of tomato-fed larvae (TP27, TP41, and TP100), there was a substantial increase in the Cox index value compared to the TP0 value, equal to 3.2. The TP41 and TP100 diets, in particular, increased by +25% and +80%, respectively. The UI data also follow an increasing trend; compared to the control values TP41 and TP100, they show an increase of +7.7% and +25%, respectively. Among the lipid quality indices related to the incidence of coronary heart disease, IA, IT, and HH did not appear to be significantly influenced by diet, except for the TP100 diet. Larvae fed with 100% tomato pomace showed IA and IT decreased by −20% and −12% compared to the control, and HH increased by +18% compared to the control.

### 3.6. Crude Protein

**Figure 3:**
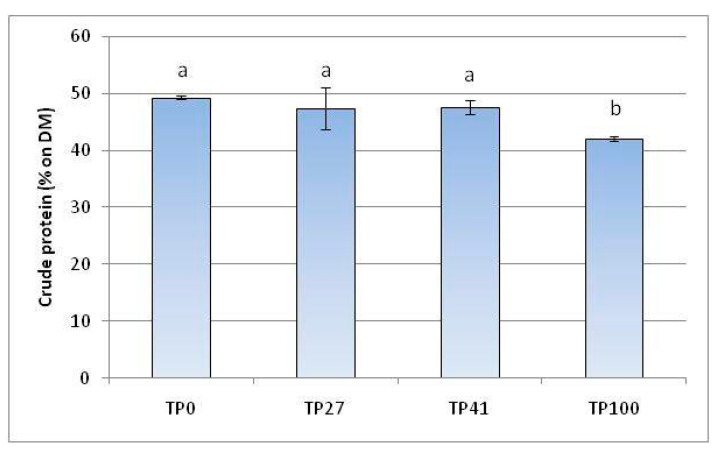
Crude protein quantification in defatted larvae fed on different diets. TP0 as control. The mean ± standard deviation values (*n* = 3) with the same letter are not significantly different at α = 0.05 (ANOVA and Tukey–Kramer HDS test).

Larvae fed on control, TP27, and TP41 diets showed similar crude protein contents (Figure 3). Their values varied from 47.3% to 49.2%, expressed on the DM of larvae defatted. In contrast, crude protein in larvae on the TP100 diet decreased by 42%.

## 4. Discussion

The nutrient composition is important for the use of by-products in diets. The dried tomato pomace used in this study is particularly poor in macro-nutrients, probably because it derives from a very efficient industrial process [28]. Its contents in protein (9.5%), carbohydrate (8.9%), and lipids (3.2%) are unfavorable in carbohydrates when compared to optimal compositions described by Morale-Ramos (20–25%, 65–75%, and 3–12%, respectively) [50] or by Kröncke and Benning (20–23%, 67–72%, and 9–10%, respectively) [12].

TP’s protein/carbohydrate ratio (p:c) is 1:0.9 and is similar to the 1:1 ratio, considered the best as it is actively regulated by adults choosing between nutritionally unbalanced diets [75]. However, these authors tested adults on synthetic diets and reported the “tendency to prioritize the regulation of carbohydrate intake over that of protein intake” [75]. More specifically, the ratio would be 1:1.6 for males and 1:1.3 for females [76]. All that would indicate the need to supplement TP with carbohydrates. However, the TP100 diet results indicate that protein and carbohydrate deficiency had a more prevalent effect on their p:c ratio. TP100 larvae had lower protein content than larvae from TP0, TP27, and TP41 isoproteic diets, in contrast to the expected maximum protein accumulation with a ratio between 1:1 and 2:1 [75].

The TP100 diet has the lowest energy value (236.6 kcal/100 g), and the TP0 diet has the highest (318.3 kcal/100 g), but they all have lower energy values than the poorest diet (353 kcal/100 g) reported by other authors [77]. The low energy value of TP is determined by the high fiber content (67.1%), considered unfavorable if more than 5–10% in diets for *T. molitor* [78].

In our study, the tested diets induced few differences in larval performance. Major and minor larval and pupal weights were obtained in the TP41 and TP100 diets, respectively; however, only the pupal weight in the TP41 diet was significantly higher. Much more important was the significant increase in larval time of development in the TP100 diet (twice as much as compared to the control). This delay in larval growth is a potential limitation to the use of pure TP due to the resulting increase in rearing costs. The increase in larval times of development can be mainly attributed to the low protein content, as there are no differences between isoproteic diets. This hypothesis agrees with the reduction inlarval times of development observed in diets richer in proteins [79].

Results on the use of diets have highlighted the significant increase in the FCR value as the dose of TP increases. This result is expected in TP100 as a remedy to compensate for the low concentration of nutrients [75]. Furthermore, the increase in FCR values in isoproteic diets suggests a positive correlation to the fiber content and a negative correlation to the energy value of the diet. Our FCR values (from 2.7 to 4.3 for TP0 and TP100, respectively) are slightly higher than some control diets, such as wheat bran and yeast (FCR = 2.3) [47], chicken feed (FCR = 1.57), and wheat bran (FCR = 2.08) [9]. It is important to point out that the FCR value (3.8) recorded in the TP41 diet is similar to 3.5 of the commercial diet for *T. molitor* used by van Broekhoven et al. [80] with similar protein values (16.5 and 17.1%, respectively).

The tested diets have SRG values in line with the seed-clearing process by-products values (2.7 to 7.2% day^−1^) [47] but are lower than the mixed diets (8.2 to 11.9% day^−1^) [12]. The particularly low value (2.5% day^−1^) of the TP100 diet is probably influenced by the long larval time of development.

The ECI values significantly decreased from 15.4% to 9.8% with the increase of the TP in the diets; they are lower than the control diet used in commercial insect rearing companies (19%) [80] and chicken feed (almost 22%) [9]. However, we find our results morecomparable to Kroncke and Benning [12] and Morales-Ramos et al. [81] for a similar mode of diet administration. Administration through “cookies” greatly reduces self-selection in mixed diets, thus reducing the possibility of self-reducing the negative impact of unbalanced diets or fibers. In this case, our ECI values fall within the range 5.5–18.4% described by Kroncke and Benning [12] and higher than the range 7–10% [81], where the two best diets have ECI values similar to the TP100 diet (with 9.8%).

The ECD values (30.0 to 65.9%) were higher compared to the values (17 to 20%) found in larval density tests [82]. The limited knowledge on ECD and the high value of the TP100 diet suggest more studies hypothesizing better conversion of the digested diet if it is poor in nutrients.

Carotenoids and fatty acid composition evidence the positive influence of tomato pomace on larval quality. The degree of accumulation of carotenoids observed in the larvae is very low compared to that contained in the substrate and feces. This reduced efficiency of larval accumulation against an evident enrichment in frass carotenoids is in agreement with other data present in the literature on mealworms fed with former foodstuffs [83] and *H. illucens* fed with agri-food by-products (ground and coarse tomato) [43]. The use of commercial β-carotene supplements administered to insects shows larval accumulation values comparable to our results [84], as well as MLW fed with leaves of *Moringa oleifera* (Lam.) [85] or with carrot pomace [86].

The lycopene content in the larvae appears to be much lower than the β-carotene content accumulated in the substrate and feces. These data arein agreement with the reduction inthe mass balance also found for other non-provitamin A carotenoids, such as zaexanthin and lutein in *T. molitor* [83] and in *H. illucens* [43] probably due to bioconversion phenomena by the insect (β-cyclase and carotene-9′,10′-monooxygenase) [87,88] or by the gut microbial community [89].

Of great interest is the observation of the effect of diet on the quantity and quality of lipids and FAs. In the MLW, lipids are second only to proteins in quantity [90]. TP0 larvae show a crude fat value (~30.8%) that is very comparable with other previously published data in which there is great variability in its concentration (from 22% to 42%) [16,60,91]. Insects, particularly MLW, have a sophisticated enzymatic kit (Elongase and Desaturase) that allows them to synthesize *de novo* fatty acids, particularly PUFAs [15,92,93].

Furthermore, they can modulate the degree of lipid accumulation and change their profile in FAs depending on the developmental stage, sex, growth environment, and especially the type of feed used [94,95,96,97,98,99].

Larvae fed with 100% TP showed a reduction in crude fat percentage compared to the control. This decrease agrees with many studies showing how caloric restriction, total carbohydrate intake in the diet, and, in particular, the addition of fatty acid and carotenoid supplements affect fat synthesis and accumulation in insect larvae and other animals [76,80,85,100,101]. Low levels of intramuscular fat are found in lamb-fed diets enriched in lycopene [102] and in pork-fed diets, rich in linoleic acid [103] and supplemented with 15% TP [104].

The fatty acid composition of TP0 larvae also confirms it as one of the most abundant sources of OA, PA, and especially LA compared to other animal sources rich in fatty acids and especially ω-6 such as chicken fat and egg yolk [105]. LA and ALA are PUFAs defined as ‘essential’ for the human body, which cannot synthesize them [106] and are essential for human health and, in particular, for preventing cardiovascular disease, one of the leading causes of death worldwide [15].

The increase in the amount of ω-3 in feeds due to TP led to a general increase in PUFAs, especially LA and ALA, and to a decrease in both OA and ω-6/ω-3 ratios in especially TP41 and TP100 diets (−45%, −70%). Diets high in the ω-3/ω-6 ratio cause an increase in PUFA and ω-3/ω-6 ratios in larvae [107] and can modulate the activity of both Δ-12 desaturase [17], which converts ω-9 oleic acids into ω-6 linoleic acids [108], and Elongase (TmElo1 and TmElo2) involved in the synthesis of PUFAs [93].

Our results also agree with other data where MLW are fed feed supplemented with linseed, grape seeds, and winery waste sludge, showing a reduction in MUFA content [15,20]. In contrast, diets with distillery by-products (grape pomace, exhausted grape marcs, grape skin pulp [20], or sunflower [109]) produced a significant increase in MUFAs and, in particular, OA, while the inclusion of olive pomace in the feed composition did not affect the FA composition of body lipids [77]. Our results also agree with improving the quality and quantity of PUFAs obtained by adding fish oil [110]. All this emphasizes how physiological mechanisms of MLW adaptation play a key role in the quality of the lipid profile of larvae on par with diets [80,83,107,111].

Incorporating TP into feeds also increased the wholesomeness of mealworms for human and animal consumption, as indicated by the lipid indices obtained. The increase in Cox index emphasizes the positive influence of diets rich in PUFAs on the stability and shelf--life of by-products obtainable from MLW [16,59,112]. In contrast, the increase in UI, comparable to some macroalgae (*Hypnea esperi*, *Gracilaria fergusonii*, *Codium vermilara*) [67], shows the strong impact of diet on increasing the percentage of high-quality PUFAs useful for reducing the risk of heart disease [113], preventing and managing type 2 diabetes, insulin resistance [114], osteoarthritis [115], and neurological disorders [116].

The absence of adverse effects on the IA, IT, and HH indices, however, makes these larvae comparable to other diets applied to MLW [17,85] and other valid novel foods such as brown seaweed, whose consumption produces the best results for human health as it has a positive effect against cardiovascular diseases [117]. It is known that the consumption of foods or meals with low IA and IT values and high HH values can have hypocholesterolemic [118] and antihypertensive effects [119] and have positive effects on the cardiovascular system [120].

Therefore, dueto the quality and quantity of its macronutrients and the presence of carotenoids in TP, the use of an optimal diet based on this by-product can support the production of mealworms and its products (flour and oil), to be used both as foods [121,122,123] and as feed [124,125,126] with high nutritional value and positive effects on human and animal health [59,127]. Great attention must be paid to the origin and traceability of by-products used in diets to avoid accumulating and biomagnifying heavy metals in larvae [128].

## 5. Conclusions

In this study, among the diets tested, the optimal diet was assembled with wheat bran (50%), tomato pomace (41%), and yeast (9%). This diet had no negative impact on larval performance and increased the content of carotenoids and polyunsaturated fatty acids. Further studies should point to replacing yeast with a cheaper protein source, such as by-products. Using pure tomato pomace further increases the content of lycopene and β-carotene in the larvae and doubles the PUFA values. However, this diet doubles the larval time of development and reduces the protein and fat content of the larvae. Therefore, the use of the latter diet is conditioned by the economic valorization of larvae with a greater health value.

## Figures and Tables

**Table 1:** Nutrient composition of by-products preliminarily conditioned ^1^.

By-Product	Dry Matter (%)	Crude Protein (%)	Crude Fat (%)	Crude Fiber (%)	Ash (%)	Carbohydrate (%)
Bran	91.2	16.7	6.5	36.1	4.2	30.2
Tomato pomace	92.1	9.5	3.2	67.1	3.9	8.9
Brewer’s spent grain	93.4	24.7	4.8	42.0	2.6	24.0
Yeast	93.0	47.6	2.4	6.8	8.0	13.8

^1^ values are reported as a percentage on dry matter.

**Table 2:** Diets composition and nutritional values ^1^ (% DM).

Diet	Bran (%)	Tomato Pomace (%)	Brewer’s Spent Grain (%)	Yeast (%)	Protein Value (%)	Carbohydrate (%)	p:c ^4^	Crude Fiber (%)	Fat (%)	Energy (kcal (100 g)
TP0 ^2^	100.0	-	-	-	16.7	30.2	1:1.8	36.1	6.5	318.3
TP27 ^3^	50.0	27.0	23.0	-	16.6	23.0	1:1.4	45.8	5.2	296.9
TP41 ^3^	50.0	41.0	-	9.0	16.5	20.0	1:1.2	46.2	4.8	281.4
TP100	-	100.0	-	-	9.5	8.9	1:0.9	67.1	3.2	236.6

^1^ calculated values; ^2^ TP0 as control; ^3^ diets with by-product blends (*w*/*w*); ^4^ protein:carbohydrate ratio.

**Table 3:** Efficiency indicators of diets tested ^1^.

Diet	FC (mg Larvae ^1^)	FCR	SRG (% day^−1^)	ECI (%)	ECD (%)
TP0 ^2^	181.1 ± 44.7 ^c^	2.7 ± 0.2 ^d^	4.9 ± 0.7 ^a^	15.4 ± 1.1 ^a^	34.6 ± 3.3 ^c^
TP27	222.5 ± 29.6 ^bc^	3.2 ± 0.1 ^c^	4.1 ± 0.4 ^b^	13.1 ± 0.5 ^b^	42.8 ± 4.5 ^b^
TP41	291.1 ± 38.5 ^a^	3.8 ± 0.3 ^b^	4.3 ± 0.4 ^b^	10.8 ± 0.7 ^c^	30.0 ± 6.3 ^c^
TP100	245.7 ± 80.3 ^ab^	4.3 ± 0.4 ^a^	2.5 ± 0.7 ^c^	9.8 ± 1.0 ^d^	65.9 ± 12.7 ^a^

^1^ Efficiency indicators: FC (feed consumption); FCR (feed conversion rate); SGR (specific growth ratio); ECI (efficiency of conversion of ingested feed); ECD (efficiency of conversion of digested feed). ^2^ TP0 as control. The mean ± standard deviation values with the same letter within columns are not significantly different (Tukey–Kramer HDS test for FC, FCR, SRG, and ECI; Pairwise multiple comparisons with Bonferroni correction for ECD) at α = 0.05.

**Table 4:** Lycopene and β-Carotene quantification ^1^.

Diet	Feed		Mealworm		Frass	
	Lycopene (ug/g)	β-Carotene (ug/g)	Lycopene (ug/g)	β-Carotene (ug/g)	Lycopene (ug/g)	β-Carotene (ug/g)
TP0 ^2^	2.7 ± 0.2 ^d^	0.3 ± 0.1 ^d^	0.1 ± 0.1 ^c^	1.4 ± 1.0 ^bc^	0.7 ± 0.0 ^d^	12.1 ± 0.3 ^d^
TP27	22.7 ± 0.8 ^c^	45.3 ± 2.0 ^c^	0.1 ± 0.0 ^c^	1.1 ± 0.2 ^c^	12.4 ± 0.4 ^c^	51.0 ± 0.6 ^c^
TP41	52.4 ± 1.7 ^b^	95.1 ± 0.7 ^b^	0.6 ± 0.3 ^b^	2.6 ± 0.8 ^b^	24.1 ± 0.3 ^b^	76.3 ± 1.2 ^b^
TP100	179.8 ± 2.7 ^a^	241.5 ± 2.5 ^a^	1.2 ± 0.3 ^a^	7.3 ± 0.1 ^a^	39.7 ± 1.6 ^a^	147.5 ± 4.6 ^a^

^1^ Values are reported on dry matter. ^2^ TP0 as control. The mean ± standard deviation values (*n* = 3) with the same letter within columns are not significantly different at α = 0.05 (ANOVA and Tukey–Kramer HDS test).

**Table 5:** Fatty acid profile of the lipid extract of MLW powder fed different diets (% TFA) ^1^.

Fatty Acid (%)		Diets			
Common Name	Lipid Number	TP0 ^2^	TP27	TP41	TP100
Caprilic acid	C8:0	n.d.	n.d.	n.d.	n.d.
Capric acid	C10:0	n.d.	n.d.	n.d.	n.d.
Lauric acid	C12:0	n.d.	n.d.	n.d.	n.d.
Myristic acid	C14:0	3.7 ± 0.0 ^a^	3.8 ± 0.0 ^a^	3.4 ± 0.1 ^b^	2.7 ± 0.1 ^c^
Palmitic acid	C16:0	15.6 ± 0.0 ^a^	14.8 ± 0.1 ^ab^	15.3 ± 0.8 ^a^	13.8 ± 0.1 ^c^
Palmitoleic acid	C16:1	1.6 ± 0.2 ^b^	4.2 ± 0.0 ^a^	1.4 ± 0.2 ^b^	1.0 ± 0.0 ^c^
Stearic acid	C18:0	2.9 ± 0.1 ^c^	3.2 ± 0.1 ^bc^	3.4 ± 0.2 ^b^	5.6 ± 0.1 ^a^
**Oleic acid**	**C18:1**	**50.** **2 ± 0.** **2 ^a^**	**44.7 ± 0.** **3 ^b^**	**42.** **9 ± 0.** **2 ^b^**	**26.** **2 ± 1.** **5 ^c^**
**α-Linoleic acid**	**C18:2n-6**	**25.** **7 ± 0.** **3 ^d^**	**28.9 ± 0.** **3 ^c^**	**32.** **6 ± 1.** **0 ^b^**	**48.1** **± 1.0 ^a^**
α-Linolenic acid	C18:3n3	0.4 ± 0.0 ^c^	0.5 ± 0.0 ^c^	1.0 ± 0.1 ^b^	2.7 ± 0.1 ^a^
Arachidic acid	C20:0	n.d.	n.d.	n.d.	n.d.
Behenic acid	C22:0	n.d.	n.d.	n.d.	n.d.
Erucic acid	C22:1	n.d.	n.d.	n.d.	n.d.
Lignoceric acid	C24:0	n.d.	n.d.	n.d.	n.d.
Σ SFA		22.1 ± 0.2 ^a^	21.8 ± 0.0 ^a^	22.1 ± 0.6 ^a^	22.0 ± 0.1 a
**Σ MUFA**		**51.** **7 ± 0.** **1 ^a^**	**48.** **8 ± 0.** **3 ^b^**	**44.** **3 ± 0.** **5 ^c^**	**27.** **2 ± 1.** **0 ^d^**
**Σ PUFA**		**26.** **1 ± 0.** **2 ^d^**	**29.** **4 ± 0.** **2 ^c^**	**33.** **6 ± 1.** **0 ^b^**	**50.8 ± 1.** **0 ^a^**
Σ UFA		77.9 ± 0.2 ^a^	78.2 ± 0.1 ^a^	77.9 ± 0.6 ^a^	78.0 ± 0.1 ^a^
PUFA:SFA ratio		1.2 ± 0.0 ^d^	1.4 ± 0.0 ^c^	1.5 ± 0.1 ^b^	2.3 ± 0.0 ^a^
MUFA:PUFA ratio		2.0 ± 0.0 ^a^	1.7 ± 0.0 ^b^	1.3 ± 0.0 ^c^	0.5 ± 0.0 ^d^
**ω6:ω3 ratio**		**64.3 ± 3.9 ^a^**	**57.8 ± 7.3 ^a^**	**32.** **6 ± 1.7 ^b^**	**17.** **8 ± 0.1 ^c^**

^1^ Values are reported on % total fatty acid (TFA); n.d. (not detectable). ^2^ TP0 as control. The mean ± standard deviation values (*n* = 3) with the same letter within the same line are not significantly different at α = 0.05 (ANOVA and Tukey–Kramer HDS test). Lines with bold characters highlight the parameters most influenced by the different diets.

**Table 6:** Lipid quality indices of TFA obtained from MLW fed different diets.

Index ^1^	Diets			
	TP0 ^2^	TP27	TP41	TP100
Cox Index	3.2 ± 0.1 ^d^	3.5 ± 0.0 ^c^	4.0 ± 0.1 ^b^	5.8 ± 0.1 ^a^
IT	0.6 ± 0.0 ^a^	0.5 ± 0.0 ^a^	0.5 ± 0.0 ^a^	0.5 ± 0.0 ^a^
IA	0.4 ± 0.0 ^a^	0.4 ± 0.0 ^a^	0.4 ± 0.0 ^a^	0.3 ± 0.0 ^b^
HH	4.0 ± 0.0 ^b^	4.0 ± 0.0 ^b^	4.1 ± 0.2 ^b^	4.7 ± 0.0 ^a^
UI	104.4 ± 0.5 ^d^	108.2 ± 0.1 ^c^	112.5 ± 1.2 ^b^	131.4 ± 1.0 ^a^

^1^ Indices abbreviations: Cox index (calculated oxidizability value index); IT (indices of thrombogenicity); IA (index of atherogenicity); HH (hypocholesterolemic/hypercholesterolemic ratio); UI (unsaturation index). ^2^ TP0 as control. The mean ± standard deviation values (*n* = 3) with the same letter within the same line are not significantly different at α = 0.05 (ANOVA and Tukey–Kramer HDS test).

## Data Availability

All data from this experiment are contained in the article.
